# The Impact of Microenvironment and Dysplasia Types on the Prognosis of Myelodysplastic Syndrome

**DOI:** 10.3390/diagnostics14232720

**Published:** 2024-12-03

**Authors:** Irem Guvendir Bakkaloglu, Itir Ebru Zemheri, Ali Hakan Kaya, Emrah Kilicaslan

**Affiliations:** 1 Pathology Department, Kartal Dr. Lutfi Kirdar City Hospital, Istanbul 34865, Turkey; 2 Pathology Department, Umraniye Training and Research Hospital, Health Sciences University, Istanbul 34668, Turkey; ebruzemheri@gmail.com; 3 Hematology Department, Medicine Faculty, Maltepe University, Istanbul 34858, Turkey; dr.alihakan@gmail.com; 4 Hematology Department, Sultan Abdulhamid Han Suam Training and Research Hospital, Health Sciences University, Istanbul 34668, Turkey; dremrahkilicaslan@gmail.com

**Keywords:** anormal localization of immature progenitors (ALIP), MDS, microenvironment, microvessel density (MVD)

## Abstract

Background: A detailed examination of bone marrow (BM) aspiration and biopsy can provide clues regarding the course of the disease as well as the diagnostic features of myelodysplastic syndrome (MDS). Methods: Our aim is to reveal the histomorphological features of MDS, investigate the impact of dysplasia types on prognosis, and highlight the importance of the microenvironment. Results: In 130 (93.5%) of 139 cases, Wright–Giemsa-stained BM aspiration slides were evaluated, and the dysplasia diversity was examined in detail. A regression analysis of dysplasia features significant for overall survival revealed that the presence of hyperlobulation in the megakaryocytic series (*p* = 0.014, odds ratio = 3.485) and the presence of an abnormal localization of immature progenitors (ALIP) (*p* = 0.010, odds ratio = 2.206) were significantly associated with poor prognosis. Additionally, an increase in the microvessel density (MVD) was found to be associated with a poor prognosis (*p* < 0.001). A multiple regression analysis identified that MVD is the most significant parameter (*p* = 0.014). Conclusions: The diversity of dysplasia in BM aspiration and biopsy does not predict MDS subtypes; however, certain cytomorphological dysplasia types can provide insights regarding survival. The microenvironment’s impact on MDS pathogenesis is undeniable, with ALIP and MVD presence and frequency being significant factors. Thus, BM histomorphological examination, beyond its diagnostic role, also offers prognostic insights

## 1. Introduction

MDS is a type of heterogeneous clonal hematopoietic disorder characterized by ineffective hematopoiesis, evidenced by morphologic dysplasia in hematopoietic cells, bone marrow failure, refractory cytopenia, and an increased risk of transformation into acute myeloid leukemia (AML) [1,2]. The diagnosis of MDS is based on a morphological evaluation of peripheral blood and bone marrow (BM), conventional cytogenetic tests, and the exclusion of secondary causes of dysplasia [3]. Although WHO classified MDS according to molecular subtypes in 2022 [4], a comprehensive histomorphological, immunohistochemical and genetic examination of BM is often required for final diagnosis and definitive classification.

Some studies have described the features of dysplasia in MDS; however, the types of dysplasia in MDS and their relevance have still not been revealed in detail [5,6].

The BM microenvironment is very important in the regulation of normal hematopoiesis. Moreover, some findings suggest that microenvironment is involved in the early stages of MDS pathogenesis, and the course and progression of the disease. In particular, the role played by increased microvessel density (MVD) in MDS prognosis, and AML transformation, is controversial [7]. Also, changes in the microenvironment can lead to the abnormal localization of immature precursors (ALIP), which can be assessed on BM biopsy. ALIP is a symptom of dysplasia, and contributes to the development of MDS in various ways through its effects on the microenvironment [8,9]. However, the relationship between ALIP, MDS features, and prognosis has not been clearly established.

In this study, we aimed to achieve the following:

(a) to detail histomorphological findings, evaluate dysplasia types and frequencies in aspiration and biopsy in MDS, and to document the indicators related to prognosis and overall survival;
(b) to evaluate the relationship between the microenvironmental factors revealed by biopsy and aspiration, especially MVD, and other histomorphological features of MDS, and their effect on prognosis;
(c) to present the relationship between the presence of ALIP, other dysplastic factors, and prognosis.

## 2. Materials and Methods

### 2.1. Case Selection

Adult (age > 17) patients diagnosed with MDS depending on BM biopsies between April 2016 and October 2022 in our pathology department were retrospectively evaluated. Patients diagnosed with MDS/Myeloproliferative neoplasm (MPN) and patients in which pathology specimens, clinical or laboratory follow-ups could not be obtained were excluded from the study.

Data regarding patients were obtained using a hematology clinic follow-up registry and a hospital and national medical database. The diagnosis of MDS was made according to the patients’ complete blood counts, peripheral smears, flow cytometry and cytogenetic tests. Diagnoses were based on the WHO 2017 MDS classification [10]. Patients diagnosed with dysplasia-related AML or those who developed AML during MDS follow-up were considered as the “group with blast rate >20%”. Cases who underwent BM biopsy or aspiration after chemotherapy or anthracycline and pyrimidine analogue treatments were excluded from the study as they may have shown treatment-related features. Our study was approved by the university ethics committee and adhered to the International Declaration of Helsinki.

### 2.2. Histochemical and Immunohistochemical Evaluation

Fibrosis in BM biopsies was evaluated with reticulin staining, and fibrosis grading was performed according to the European consensus on the BM fibrosis grading scale [11]. Iron storage was assessed using a BM aspiration smear, and the presence of iron stores was divided into 3 grades with Perl’s iron stain.

The full range of dysplasia seen in MDS was evaluated in Wright–Giemsa-stained aspiration smears and an H&E-stained BM biopsy. All histomorphological features and dysplasia variations were also compared between blast rate groups (aspiration blast rate: 0 to <5%, ≥5–<10%, ≥10–<20%, ≥20%).

BM biopsies were obtained as 3 μm sections from paraffin blocks and immunohistochemical staining was performed with CD34 (QBend/10 clone, mouse monoclonal antibody) at a 1/100 dilution (Leica), with CD117 (ep10 clone, mouse monoclonal antibody) at a 1/200 dilution (Leica), with CD20 (L26 clone, mouse monoclonal antibody) at a 1/200 dilution (Leica), with CD3 (LN10 clone, Mouse monoclonal) at a 1/500 dilution (Leica) (2GV6, rabbit monoclonal antibody) ready for use (Roche), Leica Bond-Max at a 1/200 dilution, with MPO (rabbit, plyclonal antibody) (Leica) ready for use (with Roche), and with CD61 (2f2 clone, mouse monoclonal antibody) (with Leica), ready for use (with Roche), performed automatically on the device.

Membranous or cytoplasmic CD34 and membranous positive CD117 cells were measured using an ocular ruler at 400X magnification in areas of at least 10 mm². The CD34 and CD117 cell count was expressed as a percentage of the total number of nucleated cells (9).

**Figure 1:**
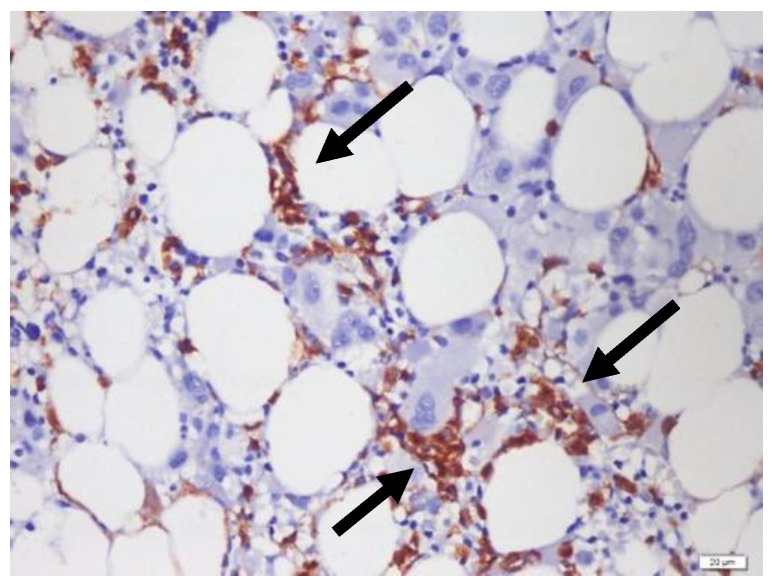
Immature precursors forming aggregates (with arrows) of three or more (ALIP) (CD34 IHC, 40×).

ALIP was defined as an aggregate of three or more myeloblast (3–5 cells) or promyelocyte (>7 cells) clusters distributed throughout the intertrabecular space. In this study, ALIP positivity was defined as at least three aggregates positive for CD34 (Figure 1), distantly localized from the endosteal surface [12].

**Figure 2:**
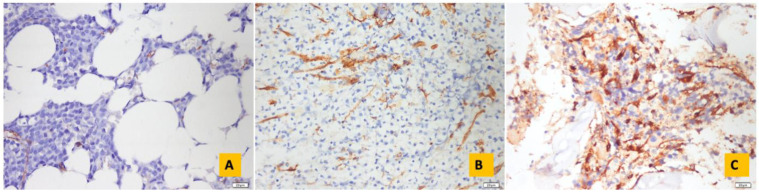
Microvessel density: (**A**) low, (**B**) medium and (**C**) high group (CD34 IHC, 40×).

For MVD, CD34-stained BM biopsy slides were first examined at 100× magnification and determined as “hot spots” on each slide at 200× magnification. In each of these “hot spots”, micro vessels (capillaries and small venules) were viewed at 400× magnification, and 10 areas were counted and averaged, as described in the literature [13]. Micro vessels were defined as endothelial cells with a transverse diameter not exceeding 10 mm (not more than 1.5 times the size of the endothelial cell nucleus), with or without a lumen, clearly separated from each other, and singly or clustered in nests or tubes. Larger vessels and areas with vessels in the periosteum were excluded [13,14]. Each slide was given an MVD degree. Accordingly, those with MDY < 10 were grouped as low; MDY 11–20 as intermediate; and MDY > 20 as high (Figure 2) [15].

### 2.3. Clinical Evaluation and Statistical Analysis

Patients who developed AML or showed MDS Excess Blast (EB) during follow-up BM biopsy after diagnosis were included in the progressive group. OS was defined as the time between the date of initial MDS diagnosis to death of any cause or to the last clinical follow-up.

Data were analyzed using the SPSS v.29.0 (IBM Corp., Armonk, NY, USA) program. The normality of the distribution of the data was checked with the Kolmogorov–Smirnov test. Descriptive statistical methods (mean ± standard deviation, median (inter quartile range) and frequency) were used appropriately. Student’s *t*-test, one-way ANOVA and Pearson’s correlation analysis were used for parametric data; the Mann–Whitney U test, Kruskal–Wallis H analysis and Spearman correlation analysis were used for non-parametric data. Statistical significance was defined as *p* < 0.05 for all values. OS was calculated in months and parameters regarding OS were assessed using Kaplan–Meier methods and the Log-Rank test. Cox’s regression analysis was used to perform multivariate analysis and identify the independent factors associated with OS for within factor-related groups (initial laboratory findings, dysplasia types, microenvironment factors and myelogram values) and in the whole patient group.

## 3. Results

### 3.1. Demographic Characteristics of the Patients

**Figure 3:**
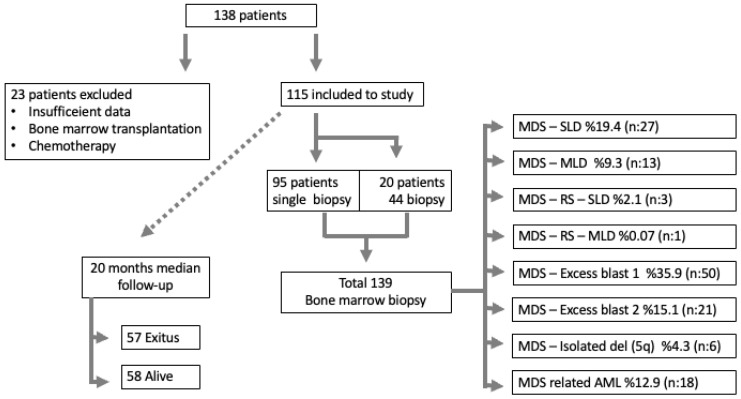
The selection of patients included in the study, the number of biopsies performed, and the MDS diagnostic subtypes.

In total, 115 patients and 139 BM biopsies were evaluated. The median age of the patients was 67 years (min: 17, max: 91, IQR: 21) and 46.9% were women (n: 54). The median survival in the study group was 20 months and 49.6% (n: 57) of patients experienced exitus. The distribution of the patients’ diagnosis according to WHO 2017 is shown in Figure 3.

### 3.2. Detailed Dysplasia Varieties

In total, 130 Wright–Giemsa-stained BM aspiration slides (eight patients lacked concurrent aspiration slides alongside their biopsy material) with corresponding H&E-stained BM biopsies were evaluated. Accordingly, dysplasia was most frequently observed in the megakaryocytic series (65.4%—n: 91), followed by the erythroid series (61%—n: 85) and granulocytic series (10.7%—n: 15).

Morphologically, nuclear budding (n: 64, 49%) was most frequently observed in erythroid dysplasia, and nuclear hypolobulation (n: 51, 36.6%) was observed more frequently in the megakaryocytic series. Among the granulocytic series, decreased granulation in the cytoplasm (n: 7, 0.5%) was observed most.

In a detailed morphological examination, the frequency of all dysplasia types and the presence of ALIP were evaluated among the blast rate groups (0–<5%, ≥5–<10%, ≥10–<20%, ≥20%). The frequency of ALIP was found to be 32% in the whole group and was significantly more frequent in cases with a blast rate ≥ 20%. However, no significant difference was shown between other dysplasia varieties among blast groups (Table 1).

### 3.3. Microenvironment Relationship

Among the blast groups, there was no correlation between the increase in blast rate and the number of B or T lymphocytes, the megakaryocytes, monocyte or lymphocyte counts, and the fibrosis degree (*p*: 0.408, *p*: 0.835, *p*: 0.648, *p*: 0.135, *p*: 0.703, *p*: 0.566, respectively).

MVD was evaluated in 98 (70.5%) patients and did not show a significant relationship with the increase in blast rate (*p*: 0.387) (Table 1). Also, no significant relationship was observed between the increase in MVD and hemoglobin levels (*p*: 0.379).

### 3.4. Patients with MDS Progression on Follow-Up Biopsy

Follow-up biopsies were available for 19 of the patients in our study. There were four (5.2%) patients who developed AML by their second biopsy, and six (5%) patients progressed to MDS EB. The demographic, initial laboratory, cytogenetic and blastic features, dysplasia diversity, microenvironment factors and other morphological features of patients who showed progression and those who did not during follow-up are summarized in Table 2. Considering patients with progression, the initial hemoglobulin value and white blood cell, neutrophil and platelet counts were significantly lower. Although MVD did not differ significantly, the presence of ALIP in initial biopsies was significantly higher in progressed cases.

### 3.5. Features Associated with OS

While a significant negative relationship was found between age and survival time (*p* < 0.001), no difference was observed in terms of gender (*p*: 0.155). Regarding the relationship between microenvironment factors and OS, the survival time decreased significantly as the number of CD3+T lymphocytes and CD61+ megakaryocytes increased (*p*: 0.003, 0.003, respectively). The PLT count (*p*: 0.005) and MVD (*p*: 0.018), among the initial laboratory findings and among the microenvironmental features, respectively, were found to be independently associated with OS. In the regression analysis where all factors were evaluated together, a higher blast rate, higher PLT count, increased age and higher MVD were found to be independently associated with a shorter OS (*p*: <0.001, 0.021, <0.001, <0.001, respectively) (Table 3).

## 4. Discussion

There are studies in the literature pointing out the relationships between dysplasia MDS types and cytogenetic mutations [6,16,17,18,19], but there is no study that details dysplasia varieties in terms of overall survival; in this respect, our study is a novelty.

In most studies, MDS is reported as a disease of the elderly population, but age is not included in existing risk scores such as the IPSS, which is used in clinical approaches. Our study revealed that age is associated with overall survival in MDS patients, independent of other disease-related parameters. On the other hand, age-related comorbidities and the decrease in expected survival, which we revealed in our study, may also cause patients’ reluctance to undertake the clinically indicated potentially toxic treatments used for many neoplastic diseases. The independent effect of age on OS in MDS patients should be considered in the treatment decision and age may be introduced as a factor affecting prognostic scores.

Cytopenia is important for both the diagnosis and prognosis of patients [1,5]. In our study, dysplasia was most frequently observed in the megakaryocytic series. As a reflection of this situation, in the peripheral blood analysis, thrombocytopenia was observed in many patients. In addition to being the most common dysplastic change, the depth of thrombocytopenia, which is a peripheral reflection of the severity of this disorder in the megakaryocytic series, is also one of the factors associated with shorter survival in our study. Parallel to this, in scores such as IPSS and IPSS-R, the platelet value is included [20]. On the other hand, the increase we showed in the number of CD61+ megakaryocytes in the BM biopsy may be due to thrombocytopenia being compensated for in these patients. The increase in CD61+ megakaryocytes was also associated with shorter survival in this study.

In our study, the most common type of megakaryocytic dysplasia was nuclear hyper-lobulation. It has been found that this dysplasia is more common in MDS/MPN and MPN, especially in essential thrombocythemia [21]. Nuclear hyperlobulation can also be seen in normal BM, but the frequency increases in MDS, including isolated del (5q) syndrome [21]. In our study, hyperlobulation was associated with micro-megakaryocytes in cases with del (5q), and was the most common megakaryocytic dysplasia seen in cases with 7q31 deletion and monosomy 7. In addition to being the most common dysplasia type, its presence is independently associated with overall survival and associated with poor prognosis. Considering this, disorders in the megakaryocytic series seem to reflect the severity of the disease and directly affect survival.

Some studies have reported the association between erythroid island topography disorder and the existence of ALIP and a poor prognosis in MDS patients [22]. In addition, in normal BM, megakaryocytes are scattered in the intertrabecular space, away from the trabeculae. Megakaryocytes approaching the trabeculae or forming clusters and aggregates are also findings that suggest dysplasia. In this study, we specifically evaluated erythroid island topography and megakaryocyte topography disorders, and no significant relationship was found between the blast rate groups or OS for either feature. This finding highlights the evaluation of bone marrow aspiration in the evaluation of dysplasia compared to the dysplasia findings that can be evaluated in biopsy.

ALIP has been identified as a histological parameter with prognostic significance in MDS [23], and may be encountered in most patients with MDS. It is associated with OS and is predictive of leukemic transformation [24]. In our study, ALIP was observed more frequently in patients with a high number of blasts. The presence of ALIP as a risk factor for the progression of low-risk MDS has been discussed in many studies and its relationship with poor prognosis has been emphasized [8,9]. In other series, a higher incidence of ALIP (up to 31%) has been reported [25]. In contrast to our and similar findings in the literature, Rios et al. reported that 38% of cases had refractory anemia with excess blasts (RAEB) and that 33% of cases had RAEBt without ALIP in a series of 120 patients analyzed on paraffin-embedded BM biopsy [23]. Also, in our study, the presence of ALIP was found to be associated with a shorter OS time.

In Germing U. et al.’s study, where they examined the detailed dysplastic cytomorphology of 3156 MDS patients, they could not show any significant difference with a specific dysplasia type, except for the MDS isolated del (5q) associated with MDS subtypes [6]. In our study, however, we did not see a statistically significant difference between MDS blast groups and dysplasia types.

It has not yet been determined whether dysplasia results from genetic or epigenetic changes caused by the hematopoietic progenitors themselves or from changes in the BM microenvironment and aging. In the pathogenesis of MDS, endothelial cells, increased vascularization, angiogenic growth factors, macrophages and monocytes are thought to affect the BM microenvironment directly or indirectly [26]. Neovascularization in the BM microenvironment is an important feature involved in the control of hematopoiesis. There are studies showing that angiogenesis is associated with MDS and AML, and thus MVD is associated with the progression of the disease [27]. In our study, no significant relationship was found between MVD and blast rate groups. Likewise, MVD is not significantly different in terms of progression. However, in our study, MVD was one of the main features that was independently associated with the overall survival of patients. Therefore, the routine evaluation and reporting of MVD may be helpful to clinicians due to its established relationship with prognosis. The fact that MVD can be routinely evaluated with the CD34 IHC marker makes this approach easier.

Studies have indicated the association of MDS with an increased monocyte count and its association with poor prognosis [28,29]. There is evidence suggesting that macrophages are potentially involved in the development of MDS; however, the cause–effect relationship and the exact mechanism by which they affect the disease have not yet been determined. Gene expression analysis of primary MDS cases has shown the altered expression of several genes, including encoding proinflammatory cytokines in macrophages [30]. In our study, we see that as the number of blasts increases, the number of monocytes tends to decrease. This may be related to the conclusion of Pang W. et al., who found that increased phagocytosis activation causes the loss of the monocyte progenitor population [31]. There are studies showing that granulocytosis and activated monocytes are important features of inflammation in the BM in MDS patients. In our study, the increase in monocytes in the myelogram was found to be a poor prognostic factor in overall survival.

The unregulated activation and clonal increase in CD8+ cytotoxic T cells in the BM have been identified as key elements in the pathogenesis of MDS [32]. In the early stages of MDS, activated cytotoxic T cells and a pro-inflammatory micro-environment contribute to polyclonal damage in hematopoiesis and promote the selection of dysplastic clones that can escape the immune response [33]. In addition, tumor-associated T lymphocyte increase may also play a role in the development of MDS and leukemia [18]. In our study, although there was no significant relationship between CD3+ T lymphocytes and CD20+ B lymphocytes among the blast rate groups, an increase CD3+ T lymphocytes was associated with worse overall survival. However, since there was no normal control group in our study, it is not possible to comment on the rate of T lymphocyte or B lymphocyte increase in the BM in MDS patients and its significance.

One of the limitations of our study was its retrospective design. The limited number of patients with a cytogenetic analysis makes related subgroup analyses difficult. On the other hand, evaluating dysplasia diversity in detail and examining its relationship with OS can be emphasized as the study’s contribution to the literature.

In summary, the diversity of dysplasia in BM aspiration and biopsy is not sufficient to provide predictive information regarding MDS subtypes or accompanying cytogenetic anomalies. The effect of the microenvironment on the pathogenesis of MDS is undeniable, and the presence and increased frequency of ALIP in MDS are both associated with overall survival. In this respect, ALIP and MVD, which can be measured with the CD34 marker used in daily practice, can be included in the pathology reports. Advanced age is an important and independent poor prognostic factor in MDS and should not be ignored in disease scoring. As a result, BM histomorphological examination, besides its pivotal role in the diagnosis of MDS, can also provide prognostic clues.

## Figures and Tables

**Table 1:** Relationships between dysplasia types and blast groups (ALIP: anormal localization of immature progenitors, HPF: high-power field, MVD: microvessel density).

	Frequency	BM Blast Count (%)				
Features (n: Number of Examined Biopsies)	% (n)	<5 (MDS)	5–10 (MDS-EB1)	10–20 (MDS-EB2)	≥20 (AML)	*p*
ALIP (n: 106)	32 (34)	8	29	20	41	<0.001
Ring sideroblast (>%15) (n: 118)	36.4 (43)	11	15.2	5.9	4.2	0.881
Erythroid topography disorder (n: 120)	36.6 (44)	39.5	38.6	47.6	23.5	0.50
Megakaryocyte topography disorder (n: 120)	34.1 (41)	52.6	45.5	47.6	23.5	0.249
Erythroid PAS+ (n: 133)	3 (4)	0	4.7	0	11.8	0.117
**Microenvironment Factors**						
CD20+ B lymphocytes (n: 80)		2 (IQR:4)	3 (IQR:3)	2 (IQR:3)	2.5 (IQR:5)	0.408
CD3+T lymphocytes (n: 81)		5 (IQR:4)	5 (IQR:5)	5 (IQR:2)	2 (IQR:9)	0.835
CD61+ megakaryocyte (n: 129)		6 (IQR:6)	6 (IQR:5)	6 (IQR:5)	3 (IQR:6)	0.648
Monocyte % (n: 106)		9.1 (IQR:3)	9.2 (IQR:2.7)	8.5 (IQR:1.8)	7.9 (IQR:1.8)	0.135
Lymphocytes % (n: 106)		11.5 (IQR:10)	12 (IQR:10)	13 (IQR:9)	9 (IQR:16)	0.703
MVD (/HPF) (n: 98)						
Group 1	24.4 (24)	10 (37%)	5 (13.5%)	5 (27.7%)	4 (25%)	0.387
Group 2	44.8 (44)	10 (37%)	18 (48.6%)	7 (38.8%)	9 (56.2%)	
Group 3	30.6 (30)	7 (25.9%)	14 (37.8%)	6 (33.3%)	3 (18.7%)	
**Erythroid dysplasia (n: 130)**						
Presence	65.3 (85)	51	70.8	63.6	72.2	0.168
Megaloblastoid change	9.4 (8)	2.3	8.3	4.5	11.8	0.473
Ring sideroblast (ever)	42.3 (36)	23.3	31.3	27.3	29.4	0.861
Nuclear bridging	7 (6)	2.3 (1)	6.3	4.5	5.9	0.834
Hyperlobulation	2 (2)	4.4	0	0	0	0.250
Asynchronous maturation	2 (2)	2.3	2.1	0	0	0.830
Abnormal chromatin condensation	10.5 (9)	4.7	6.3	4.5	17.6	0.307
Basophilic stippling	16.4 (14)	16.3	10.4	4.5	5.9	0.445
Cytoplasmic vacuolization	22.3 (19)	18.6	8.8	9.1	0	0.199
Binucleation	28.2 (24)	20.9	18.8	18.2	17.6	0.988
Nuclear budding	75.2 (64)	44.2	55.3	50	7.1	0.762
Multiple dysplasia	72.9 (62)	72	81.3	71.4	72.7	0.823
**Megakaryocytic dysplasia (n: 139)**						
Presence	65.4 (91)	74.5	62.5	68.2	44.4	0.132
Nuclear single lobulation	10.9 (10)	9.8	8.3	4.5	0	0.524
Nuclear scattered lobulation	48.3 (44)	25.5	37.5	40.9	22.2	0.353
Nuclear hyperlobulation	15.3 (14)	17.6	6.3	0	11.1	0.090
Nuclear hypolobulation	56 (51)	39.2	39.6	40.9	16.7	0.309
Micromegakaryocyte	39.5 (36)	0	0	4.5	0	0.164
Multiple dysplasia	42.4 (59)	60.5	76.7	66.7	37.5	0.187
**Granulocytic dysplasia (n: 130)**						
Presence	11.5 (15)	11.8	8.3	9.1	16.7	0.786
Auer rod	6.6 (1)	0	2.1	0	0	0.628
Hyposegmentation	20 (3)	0	2.1	4.5	5.9	0.472
Hyporanulation	46.6 (7)	4.5	4.2	4.5	11.8	0.659
Toxic granulation	33.3 (5)	4.5	2.1	4.5	5.9	0.877

**Table 2:** Characteristics differing between cases that showed progression and those that did not on follow-up biopsies (ALIP: anormal localization of immature progenitors, F: female, IQR: interquartile range, MVD: microvessel density, n: case number, * statistically significant).

Demographics and Laboratory	Progressive Cases (n:10)	Stable Cases (n:9)	*p*
Sex (F) n	4 (%40)	5 (%55)	0.656
Median age	67 (IQR:6)	63 (IQR:10)	0.656
Follow-up (months)	11 (IQR:27)	37 (IQR:24)	0.656
Hb (g/dL)	8 (IQR:4.2)	9 (IQR:1)	0.347
WBC (/L)	2.42 (IQR:1.7)	5.32 (IQR:4.8)	0.01 *
Neutrophil (/L)	1.11 (IQR:1.2)	2.245 (IQR:4.2)	1.0
Platelet (×10^3^/L)	65 (IQR:171)	216 (IQR:310)	1.0
Cytogenetics			
Any positivity of cytogenetic mutations (%)	71	0	0.167
BM biopsy features			
CD20 positivity	%2 (IQR:4)	%2.5 (IQR:1)	1.0
CD3 positivity	%5 (IQR:7)	%5 (IQR:6)	1.0
CD61 (/HPF)	8 (IQR:11)	6 (IQR:8)	0.188
MVD (/HPF)	15 (IQR:14)	18 (IQR:20)	1.0
MVD			
Group 1	%5 (n:1)	%15 (n:3)	
Group 2	%21 (n:4)	%5 (n:1)	0.253
Group 3	%15 (n:3)	%15 (n:3)	
ALIP %	66	0	0.049 *
Cell/Adipocyte	90/10 (IQR:0)	85/15 (IQR:55)	0.37
Fibrosis			
Grade 0	n:1	n:0	
Grade 1	n:3	n:4	0.283
Grade 2	n:2	n:4	
Grade 3	n:2	n:0	
Iron			
Grade 0	n:2	n:3	
Grade 1	n:3	n:0	0.161
Grade 2	n:0	n:1	
Grade 3	n:1	n:3	

**Table 3:** Univariate and multivariate analysis with Cox regression of significant parameters in overall survival (ALIP: anormal localization of immature progenitors, MVD: microvessel density, * statistically significant).

	Univariate Analysis	Multivariate Analysis			
		Within the Group		All Group	
Parameters	*p*	*p*	Odds Ratio	*p*	Odds Ratio
**Laboratory**					
Age	<0.001	<0.001 *	1.042 (1.022–1.062)	<0.001*	1.046 (1.024–1.068)
Wbc (/L)	0.028 *	0.445	0.972 (0.905–1.045)		
Neu (/L)	0.004 *	0.204	1.057 (0.970–1.152)		
PLT (/L)	0.002 *	0.005 *	0.9956 (0.993–0.999)	0.021 *	0.996 (0.992–0.999)
**BM blastic features**					
BM blast (%)	<0.001 *	0.202	1.029 (0.985–1.074)	<0.001 *	1.050 (1.020–1.081)
Myeloblast %	<0.001 *	0.648	1.013 (0.959–1.070)		
**Microenvironment Factors**					
CD3+ T lymphocyte	0.003 *	0.488	0.959 (0.851–1.080)		
CD61+ megakaryocyte	0.003 *	0.347	1.033 (0.966–1.104)		
MVD	<0.001 *	0.018 *	1.053 (1.009–1.099)	<0.001 *	1.057 (0.022–1.093)
Band cell decrease	0.019 *	0.073	0.947 (0.892–1.005)		
Monocyte increase	0.011 *	0.587	1.055 (0.870–1.270)		
**Dysplasia Types**					
ALIP	0.023 *	0.123	1.594 (0.882–2.879)		
Erythroid cytoplasmic vacuolization	0.046 *	0.096	0.417 (0.149–1.168)		
Megakaryocyte nuclear hyperlobulation	0.05 *	0.095	1.894 (0.888–4.480)		

## Data Availability

The original contributions presented in the study are included in the article, further inquiries can be directed to the corresponding author.
